# Supplementary material for: Novel multicellular prokaryote discovered next to an underground stream
Source: eLife. 2022 Oct 11;11:e71920. doi: 10.7554/eLife.71920 (PMC9555858; doi:10.7554/eLife.71920)
Supplement: Supplementary file 1. [file elife-71920-supp1.docx]

| **Supplementary file 1. Characteristics that distinguish strain HS-3 from its close phylogenetic relatives.**  ＋, Positive; −, negative; W, weakly positive. All taxa are positive for motility, catalase, acid phosphatase, N-acetyl-β-glucosamidase activity, nitrate reduction, and utilization of α-D-glucose, D-mannose, and N-acetyl-β-glucosamine. All taxa are negative for gram-staining as well as alkaline phosphatase, lipase (C14), valine arylamidase, cyctine arylamidase, trypsin, α-chymotrypsin, α-galactosidase, β-galactosidase, β-glucosidase, α-mannosidase, and α-fucosidase activity. All three strains have Q-8 as their major quinone.  **Reference data**: *J. naejangsanensis* KCTC 22633^T^ (Yoon *et al.,* 2009); *J. chitinilytica* Jchi KCTC 23701^T^ (Chen *et al.,* 2013). | | | | | |
| --- | --- | --- | --- | --- | --- |
| **Characteristic** | | | ***J. sacculi* sp. nov. HS-3^T^** | ***J. naejangsanensis*** | ***J. chitinilytica* Jchi** |
| **Shape** | | | Rods or filament | Rods | Rods |
| **Singly or in pairs** | | | Singly or in pairs | Singly | Singly |
| **Cell size** ( μm, width × length) | | | 1.0 × 1.0–1.4 in liquid  1.5×5.0–80 on solid | 0.3–0.7 × 0.7–2.5 | 0.3-0.5 × 1.0–2.0 |
| **Anaerobic growth** | | | − | ＋ | ＋ |
| **Growth at 40℃** | | | − | ＋ | − |
| **Glucose fermentation** | | | − | ＋ | ＋ |
| **Optimal pH** [on solid] | | | 6.5 [8.0-9.0] | 7.0–8.0 [6.0-9.0] | 7.0–8.0 |
| **Temperature range** (℃) [optimal] | | | 16–32 [24] | 10–40*[30] | 20–37 [25–30] |
| **Utilization of :** | | |  |  |  |
|  | | Adonitol | − | ＋ | ＋ |
|  | | Maltose | − | − | ＋ |
|  | | Xylitol | − | ＋ | ＋ |
|  | | Citrate | − | ＋ | ＋ |
|  | | Malate | − | ＋ | ＋ |
|  | | Caprate | − | ＋ | ＋ |
|  | | Cellulose | − | ＋ | − |
|  | | Tween 80 | ＋ | − | ＋ |
|  | | Glycogen | − | ＋ | **−** |
| **Enzyme activity** (by API ZYM): | | |  |  |  |
|  | Esterase Lipase (C8) | | w | w | − |
|  | Leucine arylamidase | | w | ＋ | ＋ |
|  | Naphthol-AS-BI-phosphohyderolase | | w | − | − |
|  | α-glucosidase | | − | − | ＋ |
| **DNA G + C content (mol%)** | | | 62.1 | 63.8 | 66.1 |
|  | | | | |  |

**(Continued)**

| **Fatty acid** | | ***J. sacculi* sp. nov. HS-3^T^** | ***J. naejangsanensis*** | ***J. chitinilytica* Jchi** |
| --- | --- | --- | --- | --- |
| **Straight-chain** | | (%) |  |  |
|  | C_12:0_ | 3 | — | 1.9 |
|  | C_13:0_ | 1.2 | — | — |
|  | C_14:0_ | 5.2 | 8.3 | 7.6 |
|  | C_15:0_ | 8.7 | 4 | — |
|  | C_16:0_ | 20.3 | 25.5 | 25.5 |
|  | C_17:0_ | 1 | — | 0.5 |
| **Unsaturated** | |  |  |  |
|  | C_15:1_ω5c | 0.2 | 0.4 | 0.3 |
|  | C_15:1_ω6c | 5.4 | 1.2 | 0.4 |
|  | C_17:1_ω6c | — | 6.3 | — |
|  | C_18:1_ω7c | 3.1 | — | 12.6 |
| **Cyclo** | |  |  |  |
|  | C_17:0_ | 9.7 | — | 8.2 |
| **Hydroxy** | |  |  |  |
|  | C_12:0_ 3-OH | 4.8 | 5.3 | 5.4 |
| **Summed features **** | |  |  |  |
|  | 1 | 0.5 | — | — |
|  | 3 | 33.7 | 47.1 | 29.0 |

*Fifty in the original description (Yoon *et al.,* 2009). —, ＜0.5% or not detected.

**Summed features represent groups of two or three fatty acids that cannot be separated by GLC with the MIDI system.

Summed feature 1 contains iso-C_15:1_ H and/or C_13:0_ 3-OH. Summed feature 3 contains C_16:1_ω7c and/or iso-C_15:0_ 2-OH.
